# Deep Learning-Based Subsurface Damage Localization Using Full-Field Surface Strains

**DOI:** 10.3390/s23177445

**Published:** 2023-08-26

**Authors:** Ashish Pal, Wei Meng, Satish Nagarajaiah

**Affiliations:** 1 Department of Civil and Environmental Engineering, Rice University, 6100 Main Street, Houston, TX 77005, USA; ap81@rice.edu (A.P.); wm14@rice.edu (W.M.); 2 Department of Mechanical Engineering, Rice University, 6100 Main Street, Houston, TX 77005, USA

**Keywords:** subsurface damage, convolutional neural network, Strain Sensing Smart Skin, full-field strain, damage localization, non-destructive testing

## Abstract

Structures in their service life are often damaged as a result of aging or extreme events such as earthquakes or storms. It is essential to detect damage in a timely fashion to ensure the safe operation of the structure. If left unchecked, subsurface damage (SSD) can cause significant internal damage and may result in premature structural failure. In this study, a Convolutional Neural Network (CNN) has been developed for SSD detection using surface strain measurements. The adopted network architecture is capable of pixel-level image segmentation, that is, it classifies each location of strain measurement as damaged or undamaged. The CNN which is fed full-field strain measurements as an input image of size 256 × 256 projects the SSD onto an output image of the same size. The data for network training is generated by numerical simulation of aluminum bars with different damage scenarios, including single damage and double damage cases at a random location, direction, length, and thickness. The trained network achieves an Intersection over Union (IoU) score of 0.790 for the validation set and 0.794 for the testing set. To check the applicability of the trained network on materials other than aluminum, testing is performed on a numerically generated steel dataset. The IoU score is 0.793, the same as the aluminum dataset, affirming the network’s capability to apply to materials exhibiting a similar stress–strain relationship. To check the generalization potential of the network, it is tested on triple damage cases; the IoU score is found to be 0.764, suggesting that the network works well for unseen damage patterns as well. The network was also found to provide accurate predictions for real experimental data obtained from Strain Sensing Smart Skin (S^4^). This proves the efficacy of the network to work in real-life scenarios utilizing the full potential of the novel full-field strain sensing methods such as S^4^. The performance of the proposed network affirms that it can be used as a non-destructive testing method for subsurface crack detection and localization.

## 1. Introduction

The health assessment of structures has been gaining importance, as more structures are aging, deteriorating due to environmental factors, and becoming damaged due to extreme events such as earthquakes and storms. The timely detection and accurate localization of incurred damage is important to plan a retrofitting strategy to save the structure and potentially extend its service life. Most global assessment methods indicate the presence of damage and provide its approximate location. The local assessment methods, on the other hand, scan a small portion of the structure at a time and provide accurate details of the damage which include its presence, location, and severity. The presented study deals with the latter category of methods.

The conventional method for local assessment of damage is visual inspection. This method requires expert knowledge, is prone to human errors, applicable to only reachable areas of structure, and is discontinuous monitoring. Most importantly, only surface damage can be identified by this method, while the SSD remains undetected. SSD is a concerning issue and a major contributing factor to failure in a variety of fields, such as the failure of subsurface cracks due to rolling contact for bearing steel [1]. Bearing damage due to subsurface cracks is a major contributor to wind turbine gearbox failure resulting in increased downtime [2]. SSDs such as delamination and cracks impact aerospace components, causing their failure and reduced service life [3,4]. Concrete debonding with rebars, invisible voids, and cracks in concrete are a major concern for the deteriorating state of reinforced concrete bridges [5,6,7]. SSD in steel members of steel truss bridges is responsible for causing economic loss due to repairs, replacement of bridge members, and the associated downtime [8]. SSD is found to be a critical factor in the strength degradation of brittle crystal materials [9] and ground structural ceramics [10]. SSD is also detrimental to the performance and life of silicon chips, which is a major concern for electronic and computer industries [11].

Several methods are already available in the literature for SSD detection. A ground-penetrating radar technique has been used to detect subsurface delamination in reinforced concrete pavements [12]. Infrared thermography has been used to detect concrete delamination [5] by studying the temperature difference in the sound and damaged part. A technology based on the reflection analysis of electromagnetic waves was developed to detect internal voids and debonding in concrete structures [13]. Along similar lines, reflections of an elastic wave propagating in the ground were studied to detect subsurface cavities [14]. Other technologies include extracting features from acoustic emission measurements to detect SSD in wind turbine bearings [2]. Using short pulses of sound infuses and induces frictional heating at the delamination area to detect SSD using an infrared camera [15]. The authors study the depolarization effect on the reflected light with respect to the incident light to detect SSD in ground silicon wafers in this study [11]. In all these methods, features extracted from the response of the system are used to detect the damage. These features are selected by human judgment and understanding of the subject, which may often result in the selection of sub-optimal features.

Advancements in machine learning algorithms, especially deep learning methods, allow automatic feature extraction without any human judgment. With proper training and optimization techniques, optimal features are automatically extracted for damage characterization. A study [16] provides a very nice review of computer vision and machine learning-based techniques for damage detection in concrete and steel structures. Several deep learning networks have been developed for crack detection in concrete structures [17,18,19,20], crack detection in pavement structures [21,22,23,24], spalling detection in concrete structures [25,26], and crack detection in steel structures [27,28,29]. All of the works mentioned above detect surface defects in concrete and steel. Only a handful of works are present in the literature to detect SSD, which includes concrete delamination detection [30,31] and a deep learning network [8] to detect SSD in steel members of steel truss using infrared thermography. There is a lot of scope to explore the potential of data-driven methods for SSD detection. Moreover, most of the works on SSD detection of concrete and steel structures are based on infrared thermography. Another physical quantity that correlates well with a damaged structure is the strain concentration. The location of damage corresponds to higher strains than its neighboring area, indicating the presence of damage. With the advancements in techniques to measure full-field strain maps such as S^4^ [32,33,34,35] and Digital Image Correlation [36], SSD detection methods based on strain measurement can be a viable option. There are a few studies on damage detection and localization using full-field strain information [37,38]. However, ref. [37] focuses on the surface damage of metallic structures, and ref. [38] uses strain mode shapes for localization on a global level which cannot provide a precise localization at the local level. There are no present formulations that use full-field strain information for the much more complex and difficult task of SSD localization.

In the present study, a Convolutional Neural Network (CNN) has been developed for SSD detection based on full-field surface strain data. This work tries to fill the void of deep learning networks for SSD detection and introduces strain as an indicator quantity. When working with full-field strain data, damage detection can be performed at several stages of refinement in the spatial domain using the deep CNN architecture. The basic stage is the classification of an image into a damaged or undamaged state. At this stage, no information about the shape, size, length, or number of damages is possible to retrieve. The next stage is segmenting the image into bounding boxes of damaged and undamaged regions, as shown in [8]. At this stage, the presence of damage is indicated to be somewhere within the specified rectangular region. The next stage is a more refined version of the previous stage with damage classification at the pixel level; at this stage, damage is visible with clear boundaries. The present work deals with the pixel-level classification of the damage.

The organization of this paper is as follows, First, the adopted deep CNN architecture is described. Next, the development of the dataset for training and testing of the network is discussed. Following this, the optimization algorithm is discussed and the corresponding hyperparameter tuning is shown. In the end, the testing of the trained network is performed on aluminum, steel, triple damage, and experimental datasets.

## 2. Image Segmentation Architecture

**Figure 1:**
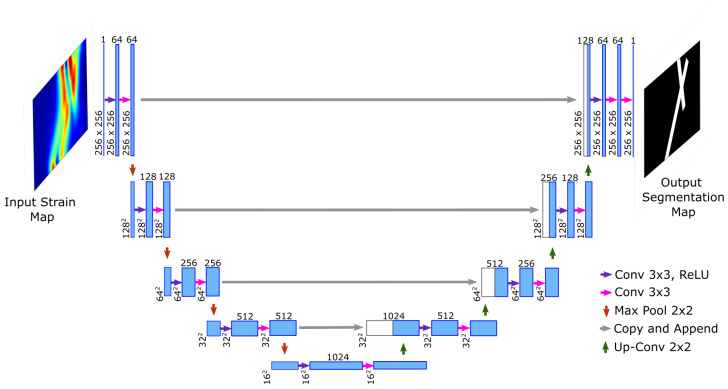
U-Net Architecture with modifications.

The U-Net ([39]) is a deep CNN architecture that was developed for the image segmentation of biomedical images. In general for training large networks, a huge amount of data are required, sometimes in thousands of annotated images. However, authors in [39] used extensive data augmentation with only 300 images to train the U-Net network. The same architecture has been adopted in this study with a few modifications (see Figure 1 for details). The network consists of a contraction path that captures the features and then a symmetric expansion path that provides localization. The original network was based on images of size 512 × 512, while in this work the input image is of size 256 × 256. The architecture consists of four steps, each in a contraction and expansion path. At each step, two 3 × 3 convolution operations are performed with zero padding to retain the same size of the input and output. Only the first convolution operation is followed by a Rectified Linear Unit (ReLU) non-linear activation, unlike the original network, where both convolution operations were followed by a ReLU layer. In the first step, the convolution operation generates 64 feature maps from the image of unit depth. In the rest of the steps, the feature maps are doubled in number from the previous step. Moving from one step to the next step, feature maps are downsampled by 2 × 2 max pool operation with a stride of 2. In the last step, there are a total of 1024 feature maps of size 16 × 16. In the expansion path, with each step, the size of feature maps is doubled while reducing their depth by a factor of 2 using a 2 × 2 transposed convolution operation. At the beginning of each step, feature maps from the contraction path are appended to feature maps of the expansion path corresponding to the same level. After appending, two 3 × 3 convolution operations are performed with zero padding. At the final step, the 3 × 3 convolution operation converts 64 feature maps to a single map which is followed by a Sigmoid activation layer for pixel-level classification.

## 3. Dataset and Data Augmentation

**Figure 2:**
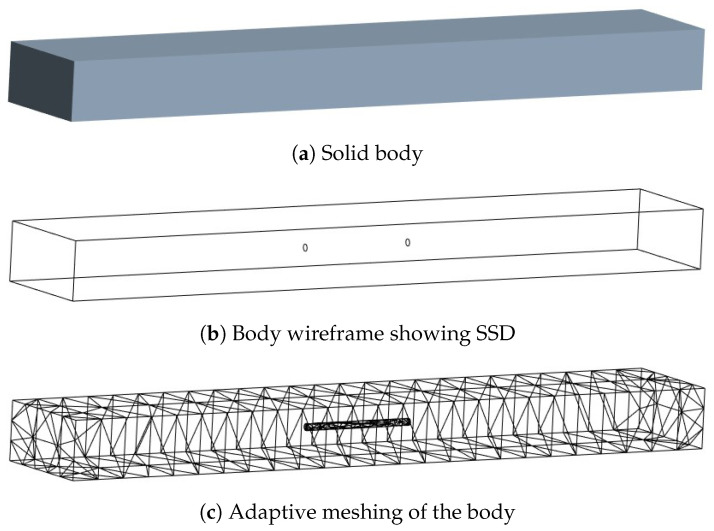
FEM design (**a**) Solid body, (**b**) Body wireframe, and (**c**) Meshed body.

**Figure 3:**
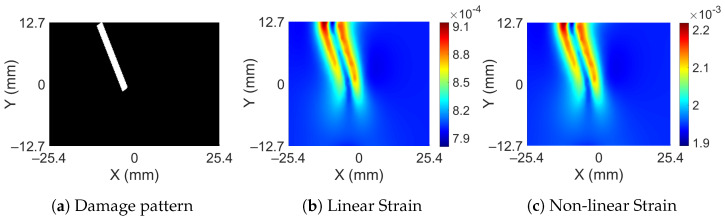
Damage pattern and corresponding surface strain distribution from linear and non-linear analysis.

**Figure 4:**
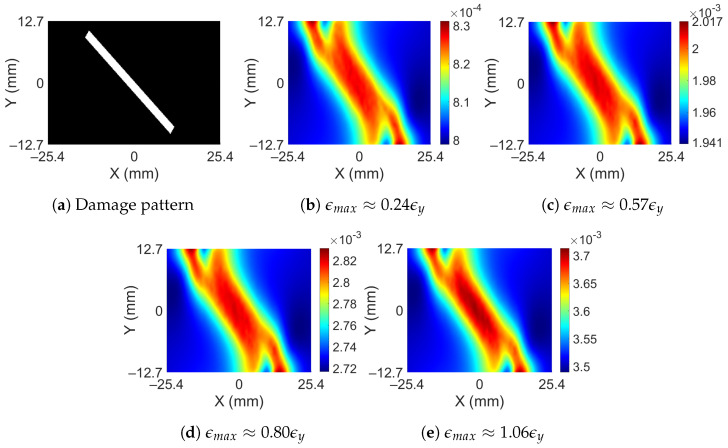
Damage pattern and surface strains for different intensity of force from non-linear analysis.

The full-field strain maps were obtained from a FE analysis of a rectangular bar using Ansys 2021 R1 (version 21.1) software; see Figure 2a. The dimensions of the bar are 152.4 mm × 25.4 mm × 12.7 mm with the assigned material properties of aluminum. The damage to the bar was provided by removing material in the shape of a cylinder at the desired depth and location, as shown in Figure 2b. Since the dimensions of subsurface damage are small as compared to the bar, adaptive meshing has been performed to generate a fine mesh around the SSD and a coarser mesh away from it, as shown in Figure 2c. Additional details about the FEM modeling can be found in the Appendix A. The bar is axially loaded and the axial strains developed at the top surface are recorded. Damage to the bar is restricted to an area within 25.4 mm on both sides of the center of the bar, and the strains are recorded within the same area. The nodal strain values obtained from the FE analysis are interpolated in a grid of 256 × 256 points. The FE analysis is performed based on the assumption that the bar remains linearly elastic; however, this may not be true if the applied force causes the bar to yield. It should also be noted that the damage is a subsurface and localized phenomenon. This means, although the redistribution of strain does take place after yielding, those effects have diminishing effects away from the damage location. Figure 3b,c shows the surface strain of a bar obtained from linear analysis and non-linear analysis for an SSD (at a depth of 3.81 mm from the top) shown in Figure 3a. In the first case, the force is kept small enough to keep the system linearly elastic (Figure 3a), while in the other case, the force is large enough to cause plastic strains to develop (Figure 3b). The magnitude of surface strains is significantly different in both cases, but the surface strain distribution looks very similar, indicating that subsurface yielding does not affect surface strains significantly. To further confirm this, another example case is taken in which a bar is subjected to incremental force until the surface strain exceeds the yielding strain. Figure 4 shows the surface strain of the damaged bar (at a depth of 5.08 mm) subjected to the different magnitudes of force. The force is gradually increased such that the average strain roughly increases by one mϵ. Except for Figure 4b, all the following figures show surface strains when yielding has occurred in the bar. Maximum surface strains ($\epsilon_{max}$) in Figure 4b are 24% of the yielding strain ($\epsilon_{y}$), while in Figure 4e, the maximum surface strain is beyond the yielding strain. If the magnitude of strains is ignored in all the cases then they look very similar; it becomes difficult to differentiate the strain distribution based on the extent of yielding in the bar. Although a small variation just above the damage can be observed in Figure 4e, the visual differences remain insignificant.

**Figure 5:**
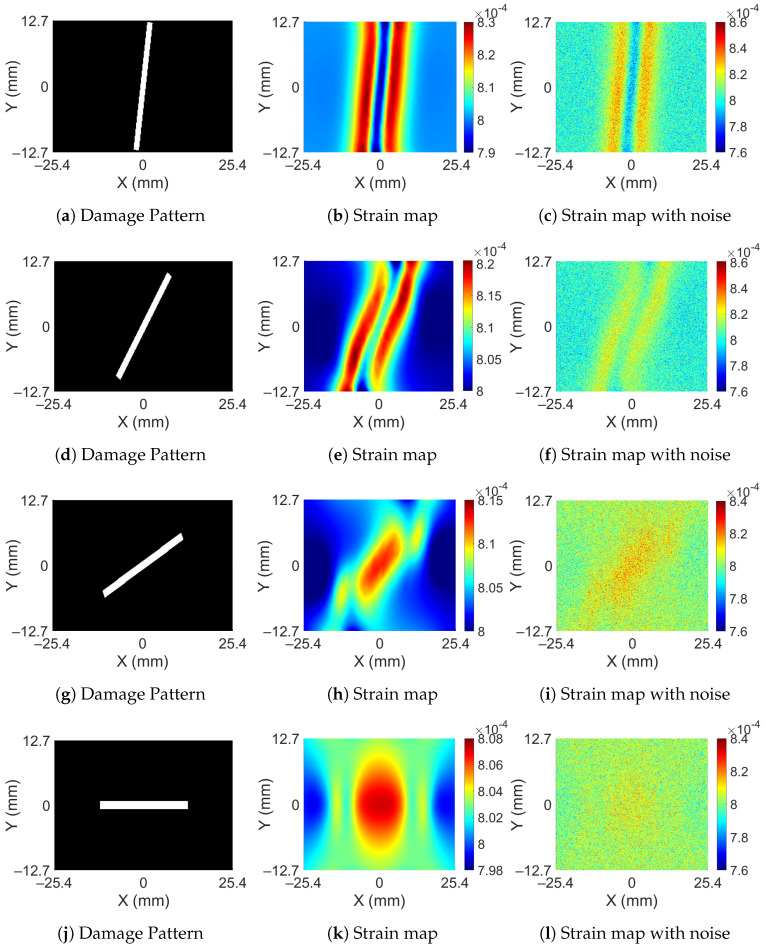
Damage patterns and their corresponding strain distribution with and without noise.

The strain variation observed on the surface of the bar is dependent on the depth at which damage has occurred and also on its orientation. As the damage occurs deeper in the bar, the spatial variation in the surface strain decreases in magnitude. Moreover, when the orientation of the damage aligns with the direction of the force, the variations show further decrements. A total of 1% Gaussian noise is added to the data for training since some amount of noise is always present in the real measurements. On adding noise, strain variations become completely lost after a certain depth, given that all the orientations of damage are allowed. Figure 5 shows surface strains for various orientations of damage which have a radius of 0.76 mm at mid-depth of the bar. Each row in the figure includes the damage orientation, noise-free surface strain obtained from the finite element method, and surface strains with 1% added noise. In the first two figures when damage is oriented perpendicular to the direction of applied force, the spatial variation is significant and is noticeable in the presence of noise. However, as the damage starts to align with the direction of force, the spatial variation in surface strain starts to decrease and it becomes hard to differentiate it in the noisy data. In the last row when damage aligns perfectly with the direction of force, it becomes difficult to tell the strain pattern without a priori knowledge of the noise-free strain pattern. Therefore, for preparation of the training data, the minimum radius of the damage is restricted to 0.76 mm, and the depth of damage is restricted until the mid-thickness of the bar.

**Figure 6:**
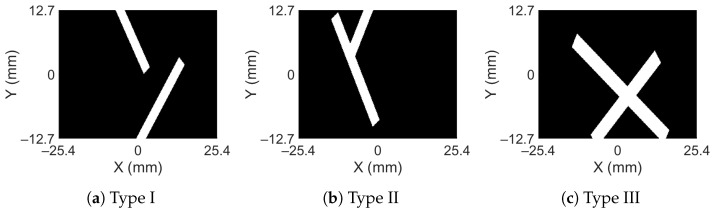
Types of double damage considered in the dataset.

When damage occurs, it is possible that it may not remain as a single damage; rather, it branches into two, or there can be two independent damages altogether. To account for multiple damages, the data includes damage formed in three ways; see Figure 6. The three types of damages are: (i) Type I—two independent damages oriented in random directions (see Figure 6a), (ii) Type II—two damages having the same root but branched at some point in a random direction (see Figure 6b), and (iii) Type III—two intersecting damages oriented in random directions (see Figure 6c). A total of 903 numerical simulations were carried out, which included 290 examples of single damage and 613 examples (the rest) from all three types of multi-damage. In all the 903 example cases, the orientation of damage is randomly assigned. The depth of the damage is also randomly assigned between the mid-depth and one radius away from the top of the bar. The radius of the damage is selected randomly from a minimum value of 0.76 mm to a maximum value of 1.52 mm.

### Data Augmentation

Although 903 examples are available to train the deep CNN network, it may still be a small number to train such a large network. Data augmentation is a powerful tool to overcome this problem which creates more training data points by augmenting the existing data. Five data augmentation strategies were adopted and applied in series, as stated below:

1. Random horizontal flip with probability = 0.5.
2. Random vertical flip with probability = 0.5.
3. Random rotation within 10 degrees with uniform probability.
4. Random shear within 10 degrees with uniform probability.
5. Random resized crop with an equal probability between a scale from 0.5 to 1.

Random rotation is restricted within 10 degrees to maintain uniqueness between the measured strain and the damage. The fifth augmentation helps add scale invariance to the network. In addition to data augmentation, data normalization is an essential and important operation in machine learning. The standard practice to normalize the data is to subtract the ensemble mean from the image and divide it by the ensemble standard deviation. One crucial thing to consider while using strain data is the huge variations that can occur in strain for the same structure. There could be a difference of an order of magnitude in strain values depending on the amount of force applied. This means if 10 N of force causes a strain of order 0.1 mϵ, then 100 N will cause 1 mϵ, while the strain pattern remains exactly the same. To make the network invariant of applied force, the individual image is normalized using an image mean and image standard deviation rather than using ensemble metrics.

## 4. Training and Hyperparameters

The adopted network takes an input image and provides a final feature map of the same size as the output. Sigmoid activation is applied to the feature map to obtain the probability of the presence of a crack at each pixel. The loss function combining Sigmoid operation and binary cross-entropy loss together is computed using the final feature map and the ground truth image, as shown:

(1) l=−[y·logσ(x)+(1−y)·log(1−σ(x))]

(2) $$\sigma \left(x\right)=\frac{1}{1+\exp(-x)}$$

where *y* is the true class, *x* is the value of the final feature map and *l* is the loss associated with the corresponding pixel. The number of pixels associated with damaged locations is much less than the undamaged locations, which creates unbalanced classes. Therefore, a positive weight is added to the positive class which in this case corresponds to the damaged pixels. The modified loss function with positive weight (say, $w_{p}$) can be written as:

(3) $$l=-[w_{p}y\cdot \log\sigma \left(x\right)+\left(1-y\right)\cdot \log\left(1-\sigma \left(x\right)\right)]$$

The loss function is minimized using Adam optimization [40] with an adaptive learning rate. The learning rate is scaled by a factor of 0.9 if the loss does not decrease by 5% for 5 epochs, and the network is run for a total of 200 epochs. Regularization using L2-penalty is added to prevent overfitting of the data. The initialization of the network is conducted using normal Glorot initialization [41]. The batch size for training is selected as 12, which is the maximum number possible for the NVIDIA RTX 2060 6 GB graphics card that was used for training.

### Hyperparameter Tuning

The dataset of 903 examples is divided into a training set, validation set, and test set by random selection. The breakup of the data is 50%, 30%, and 20% for training, validation, and testing, respectively. There are three hyperparameters in the training process, which are the learning rate (LR), the strength of regularization (SR), and the weight to the positive examples ($w_{p}$). For the tuning process, three values of LR (5 × 10${}^{-4}$, 1 × 10${}^{-4}$, 5 × 10${}^{-5}$), four values of SR (0, 1 × 10${}^{-5}$, 1 × 10${}^{-4}$, 1 × 10${}^{-3}$), and four values of $w_{p}$ (1, 2, 3, 5) were used. The optimal network was selected after performing a full grid search of 3 × 4 × 4 = 48 networks. All the networks were trained on the same training set and the accuracy of each network was calculated using the validation set. In the image segmentation problem, calculating accuracy simply by dividing correct predictions by total predictions is not a popular choice. Rather, a metric known as the Intersection over Union (IoU) score is accepted as a better indicator of the quality of the network. The IoU score is defined as:

(4) $$IoU=\frac{TP}{TP+FP+FN}$$

where TP (True Positives) are the intersection pixels of predicted and true damage, FP (False Positives) are pixels falsely predicting the presence of damage and FN (False Negatives) are the damaged pixels not detected by the network. The prediction made by the trained network uses the Sigmoid function to move the value of damaged pixels to as close as 1 and undamaged to 0. In general, the output from the Sigmoid function is simply rounded to 0 or 1 during the prediction stage. However, in the case of classification problems when a single class is found more often than another class, the threshold value can be moved from 0.5 to some other value for better prediction accuracy. Since this threshold value is not known, it can be treated as a hyperparameter and optimized using the validation set. To this end, this threshold value is also included in the grid search for locating the optimum network. The training of the network is independent of this threshold value, so only 48 networks need to be trained still. For tuning the threshold value, the validation IoU score for all 48 networks is calculated for 21 different threshold values uniformly spaced between 0 to 1. Thus, there are a total of 48 × 21 = 1008 IoU scores from which the optimum network is selected. The optimum threshold value after the analysis was found to be 0.2. The IoU score for all 48 networks for this threshold value is listed in Table 1. The IoU score for the best network with LR = 1 × 10${}^{-4}$, SR = 1 × 10${}^{-5}$, $w_{p}$ = 2, and threshold = 0.2 is found to be 0.790.

## 5. Predictions

**Figure 7:**
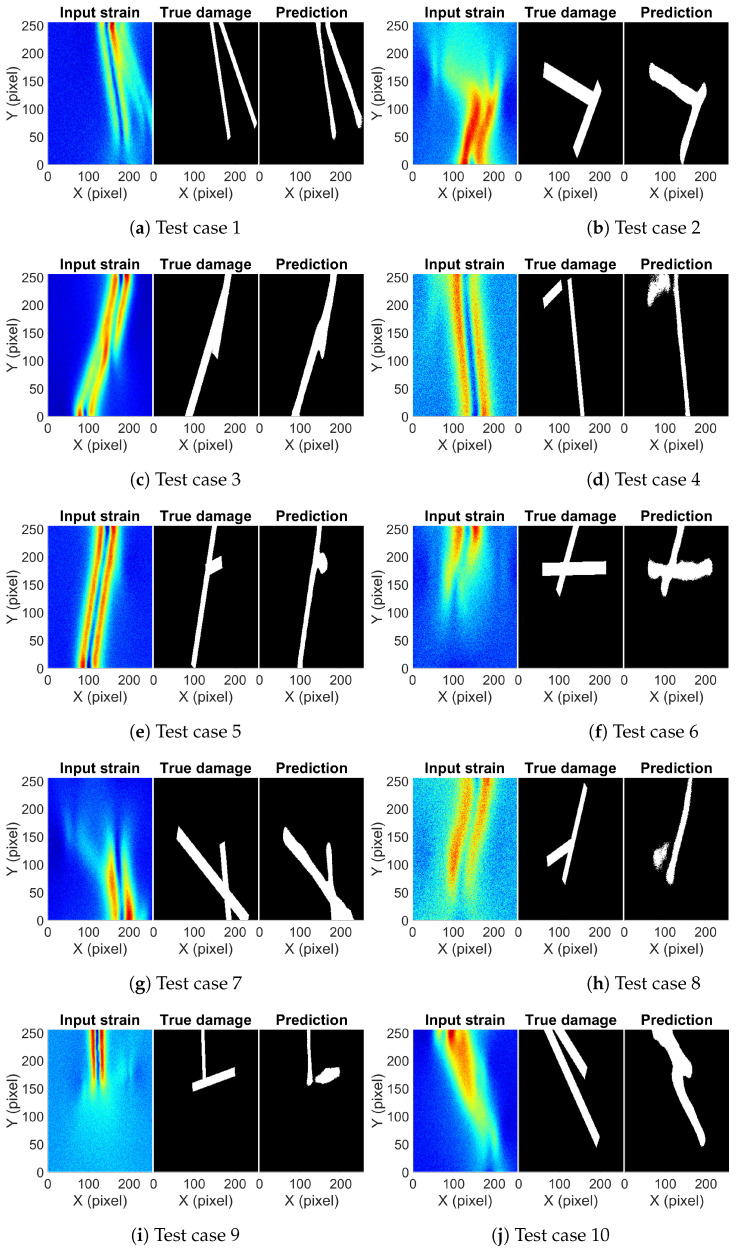
The input strain map, true damage, and predicted damage from the trained network for selected aluminum example cases.

Using the optimum network and the optimum threshold value, damage prediction was conducted for the test set which was not observed by the network. The IoU score for the test set came out to be 0.794. Figure 7 shows a few predictions on the test set along with the true damage and the input strain map. Figure 7a is the type I damage where two damages start from one end and move further apart along the width of the bar. The presence and location of both damages can be inferred by just looking at the strain map. A red strip of high strain at the top portion of the strain map indicates that damages are close enough such that strains resulting from both damages superimpose. Then, they move apart and show two patches of strain. Figure 7b is an example of type II damage, where one damage is close to vertical and the other close to horizontal. It is easy to predict the vertical damage from the strain map but not the horizontal damage. The orientation and connectivity of the two damages are difficult to predict. The red region indicating the vertical damage is only until the connection point of the two damages; beyond that, it is difficult to precisely locate the extent of the vertical damage. Figure 7c shows slant damage along the width of the bar. There is a single line of high strain in the middle with two other lines of high strain on either side of the middle line. It gives the impression that two different damages are present, but it is hard to tell whether both damages are connected or not. Both in Figure 7b,c, it is difficult to precisely find the boundary of the damages, but the trained network can accurately predict it. Figure 7d–f are examples of type I, type II, and type III damages, respectively. It is difficult to predict the presence of second damage just by looking at all these strain maps. In each case, the strain pattern indicates single near-vertical damage and no obvious strain variations indicating the presence of other damage. In Figure 7d, there is a slight greenish region at the top and left side of the dominant strain pattern, while in Figure 7e, a little bulge at the location of the second damage can be observed. In Figure 7f, on careful inspection, a small variation can be observed at the ends of the damage location. The observed variations in these three cases are subtle; to make any conclusion on the damage description will require expertise on strain patterns due to SSD. The trained network can pick up the subtle variations and correctly predict the damage location. Figure 7g–j are examples where the network shows partially accurate damage prediction. In Figure 7g, both damages are correctly picked but the gap between them at the bottom right corner is missed; the same can be observed in Figure 7h. In Figure 7i, the second horizontal damage is only partially predicted, while in Figure 7j, a part of the longer damage is missed and is merged with the smaller damage, giving an impression of the single damage. The errors in Figure 7g–j are minor, which could be attributed to the added noise to the data. It should be noted here that in cases where the network prediction has shown errors, none of those cases show any obvious strain patterns to describe the damage. It is difficult for a non-expert to accurately predict the damage by visually observing the strain map. The overall picture predicted by the network is close to the truth, showing the damaged location and shape with reasonable accuracy.

### 5.1. Prediction on Steel Dataset

**Figure 8:**
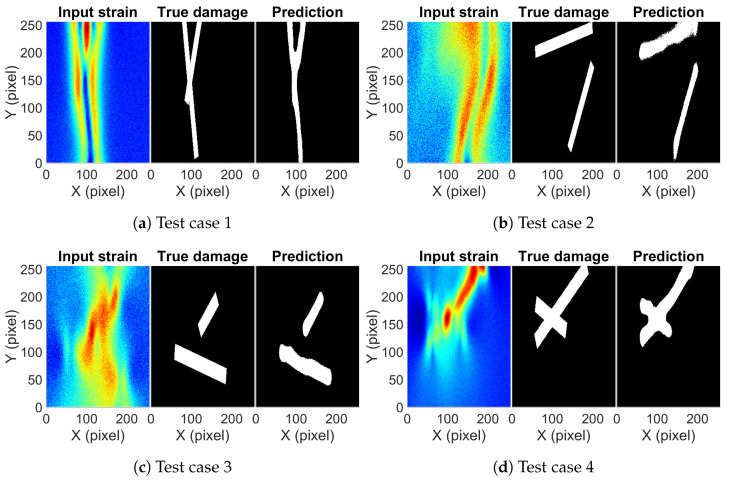
The input strain map, true damage, and predicted damage from the trained network for selected steel example cases.

The trained network on the aluminum dataset has proved to work well on aluminum test examples. Now, the performance of the network is tested on data derived from the numerical simulation of steel bars. Dimensions of the bar are kept the same as the aluminum examples. Nine example cases have been considered, each having two damages of random length, random orientation, and random diameter. Figure 8 shows predictions for four example cases. Figure 8a,d are damage cases where two damages cross each other, while Figure 8b,c are cases of two independent damages. Except for Figure 8a, there are no obvious indicators in strain maps to tell whether the two damages are crossing each other. In Figure 8b,d, the orientation and location of one damage can be predicted from the strain map; however, features of the other damage are difficult to interpret. In Figure 8c, it is very difficult to predict the orientation of both damages, as there are no clear features present that can help in predicting the correct orientation of the damage. The trained network on the other hand is able to accurately predict the location and orientation of both damages for all the example cases. It successfully identifies whether damages are crossing each other or not; in addition, the gap between both damages in Figure 8b,c is accurately predicted. The prediction of the boundary of damages is not very neat and shows some visible errors. However, the error is present in some sections of the boundary only; overall, the prediction is of an acceptable accuracy.

The IoU score for the steel test set is found to be 0.793, interestingly, which is the same as the IoU score of the aluminum test set. In the steel test set, strains were obtained from a non-linear analysis of the steel bar. Enough force was applied to yield the bar to ensure plastic strains were formed. Surface strains from the analysis of the steel bar will have the same spatial characteristics as that of the aluminum bar, since only the stiffness value is different for both materials, given that yielding does not significantly change the strain pattern. The network works well even though the magnitude of stiffness between steel and aluminum differs in orders of magnitude because of the normalization of the strain map, which makes it independent of the absolute value of the stiffness. Therefore, as long as surface strains from the non-linear response of the system show similar characteristics as the linear response (as shown before in Section 3), the trained network should work well in that case.

### 5.2. Prediction on Triple Damage Cases

**Figure 9:**
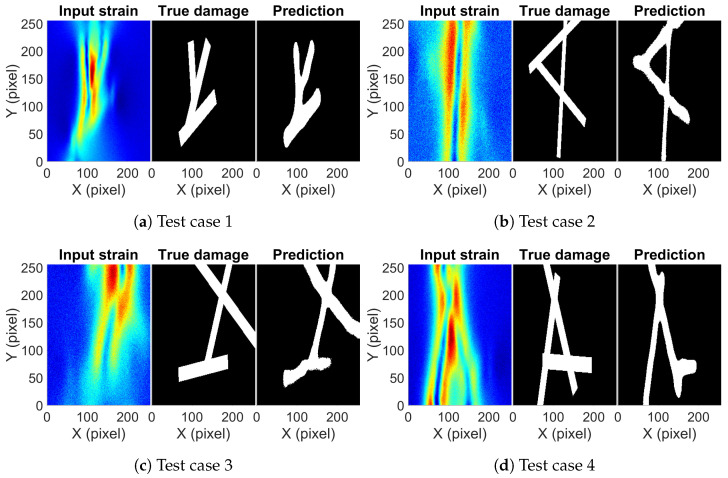
The input strain map, true damage, and predicted damage from the trained network for selected examples of triple damage cases.

The performance of the network is now checked on totally unseen damage cases. The network was trained on single and double damage cases and it did well on the aluminum test case and steel test case, which were also formed from single and double damage cases. It will be interesting to observe how well the network works on triple damage cases to obtain an estimate of its generalization potential. A total of 125 example cases were generated using a numerical simulation consisting of three damages of random length, random orientation, and random thickness. Predictions on four cases along with a true damage and strain map are shown in Figure 9. Figure 9a shows damage diverging into two damages at the bottom point and then the left leg splits again into two damages. The trained network is able to predict the correct damage pattern without any trouble. Figure 9b,c shows three damages randomly crossing each other. In these two cases, the prediction of the damage pattern is correct with some distortion along the boundary of the damage. In Figure 9d, the network predicts two of the three damages correctly but misses out on a part of the third damage. In none of the cases except Figure 9a, the strain map showed patterns directly indicating all the damage locations. It is easy to predict one of the three damages by just looking at the strain map but after that, it becomes difficult to judge, and the probability of making human error increases. The trained network does a good job at it by getting an IoU score of 0.764 for the test set of 125 example cases.

## 6. Experimental Validation

**Figure 10:**
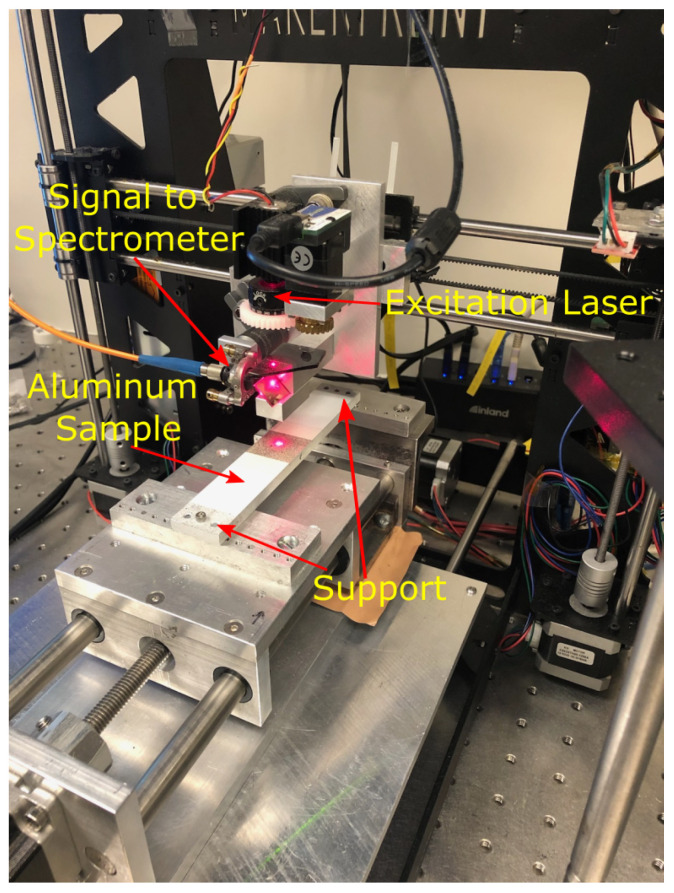
Experimental setup for testing aluminum specimen.

The network trained on numerically simulated data is now tested on the strain map obtained from the experiment on an aluminum bar that is 152.4 mm long, 25.4 mm wide, and 6.35 mm thick. The bar has a SSD in the form of a cylindrical hole along its width. The damage is 8.47 mm long starting from the edge of the plate, which is one-third of the width of the plate. The damage is provided at the mid-depth of the bar. The experimental setup in Figure 10 shows a bar fixed from both sides, except the displacement along the longitudinal direction is allowed on one side. The strain measurement is performed using S^4^. The laser head shown in Figure 10 shoots a laser at a point where the strain needs to be measured. The thickness of the laser is so small that the measured strains can be treated as point measurements. The laser head is then moved along a two-dimensional grid to obtain a full-field strain map. The measured strain map need not necessarily be in a grid of 256 × 256 points, which is the input size of our network. Therefore, the measured strains are mapped to a grid of 256 × 256 points by interpolation using cubic splines.

**Figure 11:**
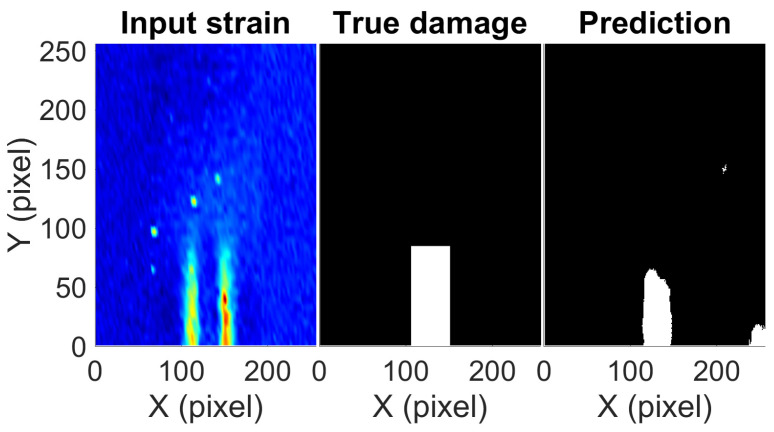
The measured strain map, true damage, and predicted damage for the experimental data obtained from S^4^.

During the experiment, the bar was stretched beyond the yielding point to allow plastic strains to form. The bar was then unloaded and a strain map was generated which measured the residual strains formed during the process. Since the location of strain concentration during loading will be the same as the location of the residual strains, the prediction of SSD can be conducted using residual strains as well. Figure 11 shows the predicted damage, true damage, and measured strain map from S^4^ for the bar. The network is able to detect the correct damage location at the center bottom region. The width and length (region) of the true damage are 3.18 mm and 8.47 mm, respectively, while the estimated damage is 2.47 mm wide and 6.54 mm long. The CNN underestimates the width of damage by just 0.71 mm and length by 1.93 mm. Given the noise in the measured data and external factors such as imperfect loading and boundary conditions causing changes in the strain pattern as compared to the numerical simulations (with ideal conditions), the relative error of 22% in both width and length estimates can be considered to be of reasonable accuracy. The network’s ability to pick up damage indicates that training based on numerically simulated data is working on real data as well. A small blob of false damage detection can be observed at the bottom right corner of the prediction in Figure 11. The reason for this is the eccentricity in the measured strains due to imperfect loading which causes strains on the right side of the damage to be slightly greater than the left side. It gives a false indication of a rather small damage somewhere on the right side. However, given the amount of noise and eccentricity in the measured strains, the majority of the damage is predicted in the region of the true damage. It is difficult to include such unpredictable conditions in the finite element modeling which assumes ideal conditions on which the network is trained. Real-life strain data for SSD is scarce; because of this, the training of the CNN was not possible on real data. However, the experiment validation shows that CNN trained on FEM data does work on real data with a possibility of a few anomalies. With the increasing availability of real-life data, transfer learning can be adopted in the future to update the proposed network with a considerably fewer number of data points to obtain a more robust network.

## 7. Conclusions

A deep CNN network based on surface strains is developed to detect SSD. The architecture of the network is adopted from U-Net with some modifications which can perform pixel-level segmentation tasks to classify them as damaged or undamaged pixels. A dataset containing 903 full-field strain data (256 × 256 points) was generated by numerical simulation of aluminum bars with diverse damage scenarios. It was shown that surface strains obtained from linear analysis had similar strain patterns with those from non-linear analysis, even when surface strains reached a near-yielding strain. Therefore, all the simulations were based on a linear analysis of the aluminum bar. Out of 903, only 452 strain data were used for training, which is a lot less than the total number of parameters in the network architecture. Therefore, extensive data augmentation was performed by random flips in the horizontal and vertical direction, random rotation, random shear, and random resized cropping. The optimum network was obtained by tuning four hyperparameters: the learning rate (1 × 10${}^{-4}$), the strength of regularization (1 × 10${}^{-5}$), the weight of positive samples (2), and the threshold value (0.2) for classification.

The physical principles in this study mainly pertain to the relationship between damage–stress–strain. The flow of information from damage to stress concentration to strain pattern is rather direct and highly correlated, which the trained neural network learns. Since the sub-surface damage causes complex surface strain patterns, the adopted convolutional neural network (CNN) learns the complex and nonlinear relationships between them and provides damage localization.

The trained network was able to segment damage in the test set for all damage types to an acceptable accuracy. The localization of damage was accurate with clear boundaries except for a few cases with minor errors. The IoU score came out to be 0.794 for the aluminum test set and 0.793 for the steel test set. The similarity in the performance even when material changes is because surface strains are less sensitive to the non-linearity introduced by subsurface yielding. As long as surface strains show similar strain patterns in the presence or absence of subsurface yielding around damages, the proposed network should work well for any material. The testing of the network on unseen triple damage cases achieved an IoU score of 0.764. This shows that the network has learned hidden and complex characteristic patterns from the surface strains and it is capable of localizing damage for unseen damage patterns. The network was also tested on experimental data where the strain was measured using Strain Sensing Smart Skin. The accuracy of prediction was reasonably good and it proved the efficacy of the network to be used on measured full-field strain data. For future improvements, updating the network using real-life/experimental data will enable it to identify the anomalies in the measurement data which should improve the robustness of the network.

## Figures and Tables

**Table 1:** IoU score for 48 networks corresponding to threshold value of 0.20.

**LR**	5 × 10${}^{-4}$				1 × 10${}^{-4}$				5 × 10${}^{-5}$			
**SR**	0	1 × 10${}^{-5}$	1 × 10${}^{-4}$	1 × 10${}^{-3}$	0	1 × 10${}^{-5}$	1 × 10${}^{-4}$	1 × 10${}^{-3}$	0	1 × 10${}^{-5}$	1 × 10${}^{-4}$	1 × 10${}^{-3}$
$w_{p}$												
1	0.73	0.76	0.75	0.29	0.78	0.78	0.76	0.78	0.76	0.76	0.76	0.71
2	0.75	0.73	0.25	0.59	0.78	0.79	0.76	0.77	0.76	0.76	0.76	0.73
3	0.26	0.59	0.62	0.25	0.78	0.76	0.76	0.75	0.76	0.76	0.75	0.72
5	0.20	0.71	0.68	0.51	0.71	0.76	0.73	0.74	0.74	0.74	0.73	0.69

**Figure A1:**
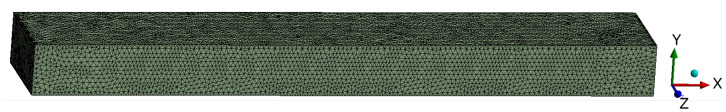
FEM mesh of the specimen.

## Data Availability

Data available on request from the corresponding author.

## Abbreviations

The following abbreviations are used in this manuscript:

CNN	Convolutional Neural Network
SSD	Subsurface Damage
SR	Strength of Regularization
LR	Learning Rate
IoU	Intersection over Union

## Appendix A

### Appendix A.1. FEM Mesh

The meshing of the specimen is conducted using 10-node solid elements which have a quadratic displacement behavior. These elements are well suited for networking irregular meshes. Since the SSD is in a random direction, shape, and size, it is better to use this type of element for meshing. Each side of the element is 0.61 mm long. Since the mesh using these elements does not produce nodes in a rectangular grid, the strain values obtained are then interpolated into a rectangular grid to input as an image.

### Appendix A.2. Material Properties

The dataset of 903 example cases used for training and testing was assigned the material properties of aluminum 6061 which is found in the engineering database of Ansys 2021 R1. The following are the material properties of the aluminum: Young’s modulus = 6.89 × 10${}^{10}$$\;N/m^{2}$, Poisson ratio = 0.33, yield strength = 2.41 × 10${}^{8}$$\;N/m^{2}$, and tangent modulus = 5.62 × 10${}^{8}$$\;N/m^{2}$.

For testing the trained network on steel specimens, the material properties of structural steel found in the engineering database of Ansys 2021 R1 were used. The steel has Young’s modulus = 2.0 × 10${}^{11}$$\;N/m^{2}$, Poisson ratio = 0.3, yield strength = 2.5 × 10${}^{8}$$\;N/m^{2}$ and tangent modulus = 1.45 × 10${}^{9}$$\;N/m^{2}$.

### Appendix A.3. Boundary Conditions

The boundary condition is fixed on one side of the bar and free everywhere else. The left face of the bar in the YZ plane is fixed by selecting the plane geometry on Ansys and setting the support conditions to a fixed support. To apply the axial force, the right face of the bar in the YZ plane is selected using geometric selection, and force is applied in the x-direction only. Since the simulation is elastic and the obtained strain maps are normalized, the magnitude of the force applied in the simulation does not matter; therefore, it is set to a fixed value for all simulations.
